# Chinese guidelines for the diagnosis and treatment of rheumatoid arthritis: 2024 update

**DOI:** 10.1515/rir-2024-0028

**Published:** 2025-01-09

**Authors:** Xinping Tian, Qian Wang, Nan Jiang, Yan Zhao, Cibo Huang, Yi Liu, Huji Xu, Yaolong Chen, Lijun Wu, Jian Xu, Hongbing Li, Liangjing Lu, Jin Lin, Lie Dai, Fen Li, Zhenyu Jiang, Zhaohui Zheng, Zongwen Shuai, Shengqian Xu, Dongbao Zhao, Miaojia Zhang, Yunlin Sun, Shengyun Liu, Caifeng Li, Pingting Yang, Mengtao Li, Xiaofeng Zeng

**Affiliations:** Department of Rheumatology and Clinical Immunology, Chinese Academy of Medical Sciences & Peking Union Medical College, National Clinical Research Center for Dermatologic and Immunologic Diseases (NCRC-DID), Ministry of Science & Technology, Key Laboratory of Rheumatology and Clinical Immunology, Ministry of Education, State Key Laboratory of Complex Severe and Rare Diseases, Peking Union Medical College Hospital (PUMCH), Beijing, China; Department of Rheumatoilogy, South China Hospital, Medical School, Shenzhen University, Shenzhen, Guangdong Province, China; Department of Rheumatology and Immunology, West China Hospital, Sichuan University, Chengdu, Sichuan Province, China; Department of rheumatology and immunology, Shanghai Changzheng hospital, the second military medical university, Shanghai, China; Institute of Health Data Science, Lanzhou University, Chinese GRADE Center, Lanzhou, Gansu Province, China; Department of Rheumatology, Xinjiang Uygur Autonomous Region People’s Hospital, Urumuqi, Xinjiang Uygur Autonomous Region, China; Department of Rheumatology and Immunology, First Affiliated Hospital of Kunming Medical University, Kunming, Yunnan Province, China; Department of Rheumatology and Immunology, Affiliated Hospital of Inner Mongolia Medical University, Hohhot, Inner Mongolia Autonomous Region, China; Department of Rheumatology, Renji Hospital, Shanghai Jiao Tong University School of Medicine, Shanghai, China; Department of Rheumatology, the First Affiliated Hospital, Zhejiang University School of Medicine, Hangzhou, Zhejiang Province, China; Department of Rheumatology and Immunology, Sun Yatsen Memorial Hospital, Sun Yatsen University, 107 Yan Jiang West Road, Guangzhou, Guangdong Province, Guangzhou China; Department of Rheumatology and Immunology, the Second Xiangya Hospital, Central South University, Clinical Medical Research Center for Systemic Autoimmune Diseases in Hunan Province, Changsha, Hunan, China; Department of Rheumatology and Immunology, First Hospital of Jilin University, Changchun, Jilin Province, China; Department of Clinical Immunology Xijing Hospital, Fourth Military Medical University, Xi’an, Shaanxi Province, China; Department of Rheumatology and Immunology, The First Affiliated Hospital of Anhui Medical University, Hefei, Anhui Province, China; Department of Rheumatology and Immunology, Changhai Hospital, The Second Military Medical University/Naval Medical University, Shanghai, China; Department of Rheumatology, the First Affiliated Hospital with Nanjing Medical University. Nanjing, JiangSu Province, China; Department of Rheumatology, Nanjing Drum Tower Hospital of Nanjing University Medical School, Nanjing, JiangSu Province, China; Department of Rheumatology and Clinical Immunology, the First Affiliated Hospital of Zhengzhou University, Zhengzhou, Henan Province, China; Department of Rheumatology, Beijing Children’s Hospital, Capital Medical University, National Center for Children’s Health, Beijing, China; Department of Rheumatology, the First Affiliated Hospital of China Medical University, Shenyang, Liaoning Province, China

**Keywords:** rheumatoid arthritis, diagnosis, treatment, guideline

## Abstract

Rheumatoid arthritis (RA) is an autoimmune disease with destructive arthritis as its main clinical manifestation, which is a major cause of disability. It is very important to formulate and update a guideline for the diagnosis and treatment of RA that adhere to international guideline development standards and can be applied to clinical practice in China. This guideline is endorsed and developed by the National Clinical Research Center for Dermatologic and Immunologic Diseases, collaborated with Rheumatologists Branch of Chinese Medical Doctor Association, Rheumatology Rehabilitation Branch of Chinese Association of Rehabilitation Medicine, Rheumatology Branch of Chinese Research Hospital Association, and Rheumatology Branch of Beijing Association of Holistic Integrative Medicine, based on grading of recommendations assessment, development and evaluation (GRADE) and reporting items for practice guidelines in healthcare (RIGHT). Evidence-based recommendation were developed for 10 clinical scenario that are most relevant to Chinese rheumatologists, aiming to improve and standardize the diagnosis and treatment of RA in China, which may finally improve the quality of life and prognosis of patients.

## Introduction

Rheumatoid arthritis (RA) is an auto-immune disease with destructive arthritis as its main clinical manifestation, which has a peak age of onset of 45 to 60 years but may occur at any age.^[1]^ Epidemiological surveys showed that RA has a global incidence of 0.5%–1% ^[1]^ and an incidence of 0.42% in mainland China. Accordingly, there are currently more than 5 million RA patients in China,^[2]^ with a male-to-female ratio of approximately 1: 4.^[3,4]^ Although the etiology and pathogenesis of RA have not yet been fully elucidated, it has been clarified that the basic pathological changes of RA are synovitis and pannus formation, which gradually cause destruction of joint cartilages and bones, eventually leading to joint deformity and function loss.^[5]^ RA is a highly disabling disease and an important cause of disability in Chinese population, and its disability rate increases as the course of disease extends.^[6,7]^ In addition, RA may be complicated by pulmonary disorders, cardiovascular/cerebrovascular disorders, osteoporosis, and malignancies,^8,9,10^] which not only causes a decline in the patients’ physical function, quality of life, and social participation, but also imposes a huge economic burden on the patient families and the society.^[11,12]^

In recent years, multiple international rheumatology academic organizations including the American College of Rheumatology (ACR), European Alliance of Associations for Rheumatology (EULAR), and Asia Pacific League of Associations for Rheumatology (APLAR) have updated their guidelines or recommendations for the diagnosis and treatment of RA,^[13,14,15]^ and the Chinese Rheumatology Association has also updated the guideline for the diagnosis and treatment of RA in 2018.^[16]^ However, with the continuous update of therapeutic drugs for RA, more and more new drugs have been approved for treatment of RA in China, while epidemiological and clinical research evidence of RA in the Chinese population is rarely included in international RA guidelines, the clinical diagnosis and medication prescription habits of foreign rheumatologists are different from those of Chinese rheumatologists. In addition, the specialty setting of rheumatology in China and reimbursement policy are also significantly different from those of foreign hospitals and countries. Therefore, revising and updating the Chinese guideline for the clinical diagnosis and treatment of RA is very important in improving the ability of clinicians engaged in RA-related diagnosis and treatment in rheumatology, internal medicine, and orthopedics departments, especially physicians in primary healthcare institutions, to correctly diagnose and treat RA, strengthening patient education, and improving the level of RA diagnosis and treatment in China. Under this background, the National Clinical Research Center for Dermatologic and Immunologic Diseases (NCRC-DID) (Peking Union Medical College Hospital), as the organizer and initiator, in conjunction with the Rheumatologists Branch of Chinese Medical Doctor Association, Rheumatology Rehabilitation Branch of Chinese Association of Rehabilitation Medicine, Rheumatology Branch of Chinese Research Hospital Association, and Rheumatology Branch of Beijing Association of Holistic Integrative Medicine, in accordance with the methods and steps formulated by evidence-based clinical practice guidelines, based on the current best evidence, combined with the experience of clinicians and the preferences and values of Chinese patients, and balancing the benefits and risks of intervention measures, updated and revised the “2018 Chinese guideline for the diagnosis and treatment of rheumatoid arthritis”, forming the “2024 Guideline for the diagnosis and treatment of rheumatoid arthritis”.

## Method

1. Guidelines sponsors: This guideline is endorsed and developed by the NCRC-DID (Peking Union Medical College Hospital). The development of this guideline was launched on 09 Jun 2023 and finalized on 07 Mar 2024.
2. Guideline development group: A collaborative and multidisciplinary team was meticulously assembled for the purpose of developing this guideline and experts in the panel are mainly from the Department of Rheumatology and Immunology and Evidence-Based Medicine. According to the work they contributed to, they are divided into the guideline expert panel and evidence evaluation group. The guideline expert panel participates in Delphi consensus, is mainly responsible for giving suggestions and comments and reviewing the drafts. The role of the evidence evaluation group includes conducting comprehensive evidence retrieval, evaluation, grading, summarizing recommendations. The drafts are composited by some of the expert panel jointly worked with evidence evaluation group. All the members of the development group completed a mandatory Conflict of Interest Disclosure form, thereby affirming the absence of conflicts of interest pertaining to this guideline.
3. Guidelines registration and plan writing: This guideline was registered on the Practice guideline REgistration for transPAREncy (PREPARE-2023CN490) on 12 Jul 2023. This guideline is an update of 2018 Chinese Guidelines^[17]^ for the Diagnosis and Treatment of Rheumatoid Arthritis. The updates of this guideline strictly follows the guidance for updating clinical practice guidelines,^[16,18]^ the World Health Organization (WHO) handbook for guideline development in 2014^[19]^ and Guiding Principles for Formulating/Revising Clinical Diagnosis and Treatment Guideline in China (2022 Edition),^[20]^ and refers to the items from Reporting Items for Practice Guideline in Healthcare (RIGHT)^[21]^ and Reporting Items for Updated Clinical Guidelines.^[22]^
4. Guideline users and target population: The intended users of this guideline are rheumatologists, orthopaedic surgeons, general practitioners, clinical pharmacists, radiologists and healthcare professionals that may engage in the treatment and management of RA. The guideline primarily targets patients with RA.
5. Selection and determination of clinical questions: Based on the clinical questions in the 2018 Chinese guidelines for the diagnosis and treatment of rheumatoid arthritis,^[16]^ 67 experts were involved to collect and expand the questions again. After discussion by the expert panel, a total of 10 key clinical questions were selected for this guideline.
6. Evidence retrieval: The Evidence Evaluation Group meticulously deconstructed the 10 identified clinical questions into their respective Population, Intervention, Comparison, and Outcome (PICO) components before embarking on an extensive search process. (1) Multiple comprehensive databases were searched, including Pubmed, Cochrane Library, China National Knowledge Infrastructure (CNKI) and Sinomed. The research type mainly included systematic review or meta-analysis, network meta-analysis, randomized controlled trial (RCT), cohort study, case-control study, case series, epidemiological surveys and other original studies, which were retrieved from January 1, 2018 to December 31, 2023. (2) search was performed on RA-related guidelines and consensuses *via* British National Institute for Health and Clinical Excellence (NICE), ACR, EULAR and APLAR and other official websites, as well as MEDLINE and China National Knowledge Infrastructure database. (3) Google Scholar and other websites were searched for supplementation.
7. Evaluation and grading of evidence: The evidence evaluation group adopted a measurement tool to assess systematic reviews (AMSTAR)^[23]^ for the risk of bias assessment of included systematic reviews, meta-analyses and network meta-analyses., Cochrane tool risk of bias (ROB, for RCT),^[24]^ quality assessment of diagnostic accuracy studies (QUADAS-2, for diagnostic accuracy studies),^[25]^ Newcastle-Ottawa Scale (NOS, for observational studies) were adopted for assessing methodological quality of original studies of corresponding types,^[26]^ respectively. Two investigators independently performed the assessments, and any discrepancies were resolved through discussion or by consulting a third investigator. The Grading of Recommendations Assessment, Development and Evaluation (GRADE) approach was utilized to grade the evidence and formulaterecommendations (Table 1).^[27,28,29,30]^
  **Table 1:** Grading of Recommendations Assessment, Development and Evaluation (GRADE)

GRADE rating	Description
Quality of evidence	
High (A)	The authors have a lot of confidence that the true effect is similar to the estimated effect
Medium (B)	The authors believe that the true effect is probably close to the estimated effect
Low (C)	The true effect might be markedly different from the estimated effect
Very Low (D)	The true effect is probably markedly different from the estimated effect
Strength of recommendation	
Strong (1)	The desirable effects of an intervention clearly outweigh the undesirable effects, or clearly do not
Weak (2)	The trade-offs are less certain—either because of low quality evidence or because evidence suggests that desirable and undesirable effects are closely balanced
8. Formation of recommendations: The recommendations were formulated by the Expert Panel based on the evidence summarized by the evidence evaluation group. The preferences of Chinese patients, as well as the costs and benefits of the interventions, were taken into consideration. Two rounds of Delphi recommendations surveys were conducted on Jan 19, 2024 and Jan 29, 2024. A total of 116 feedback suggestions were received. Then consensus was reached and further modification to the draft was made based on the expert panel discussions. The draft of the guidelines was finalized after the final meeting for discussion held on 07 Mar 2024.
9. Update of guidelines: A plan for proactive approach to guideline updates is made with a timeframe of 5 years for revision. The updates will adhere to international guideline update requirements and guideline.

## Recommendations

***Recommendation 1: Early diagnosis has a significant impact on the treatment and prognosis of RA, so clinicians need to make the diagnosis promptly based on patient’s clinical presentations, laboratory tests results, and imaging examinations (1A). The 1987 ACR and 2010 ACR/ EULAR classification criteria for RA are recommended as the reference for RA diagnosis (2B)***

Early diagnosis should be based on patient’s clinical presentations, results of laboratory tests and imaging examinations. Emerging evidence have shown that early diagnosis and treatment can reduce the incidence of joint damage and disability and ultimately improve prognosis.^[31,32,33,34]^ Currently, there are 2 international classification criteria that can be used to help diagnosing RA. There are some limitations of the 1987 ACR classification criteria for identifying early RA.^[35,36,37]^ The 2010 ACR/EULAR RA classification criteria can identify early RA patients among inflammatory arthritis patients with synovitis. 2010 ACR/EULAR RA classification criteria can assure patients to be diagnosed, so can be treated with disease-modifying anti-rheumatic drugs (DMARDs) at early stage, which may delay disease progression. Numerous studies have shown that the 2010 ACR/EULAR classification criteria have a higher diagnostic sensitivity for early RA compared with the 1987 ACR classification criteria (72.3% *vs*. 39.1%) especially for elderly patients.^[34,38,39]^ However, 2010 ACR/ EULAR classification criteria has been shown to have a lower diagnostic sensitivity for seronegative RA patients,^[40,41]^ who are negative for both rheumatoid factor (RF) and anti-citrullinated peptide antibodies (ACPA) while imaging examinations such as musculoskeletal ultrasound or magnetic resonance imaging (MRI) can assist in the diagnosis of sero-negative RA. The specificity of the 2010 classification criteria was lower than that of the 1987 criteria (83.2% compared to 92.4% in the 1987 criteria), especially in elderly patients.^[39]^ If the 2010 classification criteria are applied to all patients with arthralgia, some patients with nonspecific arthritis may be misdiagnosed as RA, while the 1987 criteria have a better predictive value for bone erosion.^[34]^

In summary, the diagnosis of RA should be based on the combination of the clinical manifestations, laboratory and imaging examinations. The classification criteria in 1987 and 2010 have their own advantages in the diagnosis of RA. Clinicians can refer to both criteria as references to make accurate diagnosis of RA based on specific characteristics of Chinese RA patients. Early diagnosis of RA facilitates early treatment and delays disease progression.

***Recommendation 2: We recommend that clinicians select the most suitable imaging modalities such as X-rays, ultrasound, computed tomography (CT), and MRI (2B) when conditions permit, based on the signs and symptoms of the patient (2B)***

Imaging examinations are effective tools to assist clinicians to make the diagnosis of RA. The value for diagnosis and disease monitoring as well as the advantages of various imaging modalities are listed in Table 2. Evidence-based recommendations for selecting imaging modalities for RA diagnosis were issued in both 2013 EULAR recommendations for the use of imaging of the joints in the clinical management of rheumatoid arthritis and 2018 Chinese guidelines for the diagnosis and treatment of rheumatoid arthritis.^[16,42]^ In addition, there are considerable differences in the availability of imaging equipment and technology in different regions of the country, therefore, clinicians should choose the most suitable diagnostic imaging modalities available locally to assist in making the diagnosis.^[42,43,44,45,46,47]^

**Table 2:** The value of imaging modalities in the diagnostics, follow up, and monitoring of RA

Image modalities	Applied situations	Advantages	Disadvantages
X-Ray	X-ray is the most commonly used imaging modality for the diagnosis and disease progression assessment of RA; Routine X-rays presentation may be normal in patients with RA whose disease course is less than half year.^[42]^	Low cost; Accessibility is very high	3D lesion shown in 2D image. Exposure to radiation. Low sensitivity to early bone damage.
Ultrasound	Can detect tissue inflammation at earlier phase than physical examination and X-rays;^[42,43]^ In early RA, ultrasound findings of tenosynovitis and synovitis can assist in the assessment and prediction of radiographic progression, but do not predict therapeutic efficacy;^[44]^ Can be used for the assessment and monitoring of relapses;^[43]^ Can be used to guide joint aspiration and treatment. ^[42]^	Medium in cost.; No radiation exposure; Can detect inflammation and early structural damage to bone and cartilage; Can be used to help in monitoring disease relapses.	Operator-dependent; Less sensitive to changes in large, deep located joints.
CT	CT is valuable particularly for large joint lesions and lung disease, but CT cannot detect active inflammation in synovitis, tenosynovitis, *etc*.^[42,45]^	Can detect bone Erosion of large joints. Can detects complicated lung disease.	Relatively high in cost. High radiation exposure. Cannot detect active inflammation.
MRI	Is the most sensitive tool to detect early RA lesions. It can detect early joint lesions, including thickening of synovium, bone marrow edema, and bone erosions.^[42]^ Can detect inflammation, so it can be used to identify subclinical inflammation and predict potential progression to RA in patients currently with current undifferentiated arthritis.^[42]^ Bone marrow edema has been shown to be one of the powerful independent predictive factors for imaging progression and but cannot predict clinical response.^[43,46]^ Can detect persistent inflammation in synovium and bone after clinical manifestations were improved, However, it is not recommended to be the only criteria for change of treatment.^[46]^	High sensitivity; No radiation exposure. High sensitivity in detecting deep located or complicated joints.	High in cost; Not widely accessible Time duration for examination is long; Only one site can be visualized per examination.
Special imaging modalities	FDG PET/CT can detect inflammation in joints and tissues and is helpful in the differential diagnosis of arthritis;^[42]^ Changes in FDG PET/CT correlate with disease activity in RA but cannot be used as a routine monitoring tool.^[47]^	It can not only assess inflammation of multiple joints throughout the body, but can also semi-quantify the severity of inflammation.	High in cost; High radiation exposure; Limited accessibility; Time duration for examination is long.

RA, rheumatoid arthritis; FDG, fluoro-2-deoxy-D-glucose; PET, positron emission tomography; CT, computed tomography; MRI, magnetic resonance imaging.

***Recommendation 3: The principles of management of RA are early, standardized treatment, regular monitoring and follow up (1A); the goal of RA treatment is to achieve disease remission or low disease activity, that is, to treat the disease to the target. The overall goal of treatment is to control the disease activity, reduce disability, and improve patients’ quality of life (1B)***

The joint lesions of RA are caused by synovial inflammation caused by inflammatory cell infiltration and release of inflammatory factors.^[48]^ To inhibit the production of cytokines and their effects as early as possible can effectively prevent or minimize the destructions in joint synovium and cartilage.^[49]^ Therefore, timely treatment should be carried out as soon as the diagnosis is made. Indeed, studies have shown that irregular use of DMARDs is one of the independent risk factors for joint function limitation in RA patients.^[6]^

Although RA is not curable, the treat-to-target strategy is effective in alleviating symptoms and controlling the disease progression. Treat-to-target is referred to treat to achieve clinical remission or low disease activity.^[50]^ Clinical remission is currently defined as follows: 28 joint disease activity (DAS28) ≤ 2.6, or clinical disease activity index (CDAI) ≤ 2.8, or simplified disease activity index (SDAI) ≤ 3.3. Low disease activity can be the alternative treatment target, *i.e*., DAS28 ≤ 3.2, CDAI ≤ 10 or SDAI ≤ 11. However, it should be noted that there are limitations in the disease activity evaluation tools and studies have shown that RA patients with swollen joints can still suffer further joint damage even when their DAS28 score is less than 2.6.^[51]^ In 2011, the ACR and EULAR proposed Boolean remission criteria, which included tender joint count, swollen joint count, C-reactive protein (CRP) level, and patient global assessment were all ≤ 1.^[52]^ Due to its high specificity and the ease of implementation, the criteria has been gradually adopted in clinical practice. Nevertheless, remission rate based on Boolean 1.0 criterion is low.^[53]^ Studies have shown that the severity of the disease and synovitis may be overestimated when using Boolean 1.0 criteria.^[54,55]^ Therefore, the ACR and EULAR updated Boolean response criteria (Boolean 2.0 criteria) in 2023 with a change in patient global assessment (PtGA) threshold from ≤ 1 to ≤ 2.^[56]^ In addition, it should be noted that the efficacy of biological DMARDs (bDMARDs) or targeted synthetic DMARDs (tsDMARDs) may be overestimated when evaluating with composite measures (*e.g*., DAS28 or SDAI) including acute phase reactants.^[57]^ Clinicians should choose the appropriate evaluation criteria according to their real practice situation.

***Recommendation 4: For patients who are treatment naïveor who do not achieve the treatment target, we recommend to assess their disease activity once every 1–3 months (2B); For patients who have reached the treatment target, we recommend to monitor their disease activity once every 3–6 months (2B)***

A systematic review in 2019 comprehensively analyzed 22 guidelines on the treatment of RA, of which 18 recommend regular assessments using a variety of clinical assessments.^[58]^ A cohort study have found that every-3-month assessment of disease activity and following a treat-to-target strategy lead to higher rates of remission in patients with RA.^[59]^ In a RCT, monitoring and adjusting the medication regimen monthly resulted in better treatment response compared to the regimen done once every 3 months.^[60]^ For RA patients on initial therapy, we recommend to assess their disease activity monthly, taking into account the onset time of effect of DMARDs and the adverse reactions; the monitoring frequency for patients who have reached the therapeutic target can be adjusted to be once every 3–6 months.

***Recommendation 5: The choice of treatment should be based on a comprehensive consideration to the disease activity and poor prognostic factors, as well as extra-articular manifestations and comorbidities (1B)***

Evaluation of disease activity and poor prognostic factors can provide important clues to clinicians to adjust treatment plans and select appropriate medications. As mentioned above, DAS28, SDAI and CDAI, which are composite criteria composed of swollen joint count, tender joint count, erythrocyte sedimentation rate (ESR), and CRP level, can reflect the disease activity accurately so to provide basis for setting treatment targets and adjustment of treatment regimen. In addition, multiple prognostic studies and predictive models have shown that RF and ACPA are predictive factors for joint damage progression in addition to disease activity^[61,62,63]^; however, it should be noted that these autoantibodies do not directly correlate with disease activity, and the reduction in RF and ACPA titer shall not be chosen as a treat target. Disease activity and poor prognostic factors can help physicians to select the optimal treatment regimen.

Furthermore, RA patients, especially patients who have a long-term disease course and are not well controlled, may be at the risk of extra-articular manifestations, including rheumatoid nodules, interstitial lung disease, pleurisy, pericarditis, vasculitis, peripheral neuropathy, keratitis, scleritis, and Felty’s syndrome.^[8]^ RA patients with extra-articular manifestations will develop more complications and carry poorer prognosis, especially when complicated with severe interstitial lung disease.^[8,64,65,66]^

Studies have shown that compared with the general population, RA patients have increased risks of cardiovascular/ cerebrovascular disorders,^[67,68,69]^ osteoporosis and fragility fracture,^[70]^ sarcopenia,^[71,72]^ malignancy,^[73]^ and tuberculosis. ^[74]^ These comorbidities will also cause an adverse impact on disease activity, joint damage progression, and treatment regimen of RA patients.^[8,75,76]^

Therefore, clinicians should evaluate every patient’s condition comprehensively and monitor the disease activity, poor prognostic factors, extra-articular manifestations, and comorbidities regularly, in order to develop the most appropriate treatment regimen and adjust the treatment plans accordingly.

***Recommendation 6: Once RA is diagnosed, conventional synthetic DMARD therapy should be initiated as early as possible (1A); we recommend methotrexate (MTX) monotherapy as the first-line therapy, but other conventional synthetic DMARDs should be considered when MTX is contraindicated or not tolerated (1B)***

Once RA is diagnosed, conventional synthetic DMARD therapy should be initiated as early as possible, which will help alleviate clinical symptoms, delay radiographic progression, and improve prognosis. At present, MTX monotherapy is recommended as the first-line therapy by international guidelines.^[13,14,15]^ MTX is usually given at an oral dosage of 7.5 to 20 mg/week and the dose should be adjusted timely according to disease activity, treatment response, and adverse reactions.^[77,78]^. Folic acid supplementation at 5 mg/week is recommended for patients receiving MTX to alleviate adverse reactions.^[79,80]^ Sulfasalazine or leflunomide is recommended to use in the patients who are contraindicated or intolerant to MTX.^[14,81,82,83,84]^ The recommended doses for sulfasalazine is 3 g per day, and the dosage of leflunomide is 20 mg per day. The mechanism of action, dosage, and adverse reactions of conventional synthetic DMARDs (csDMARDs) for the treatment of RA can be referred to Table 3.

**Table 3:** csDMARDs in the treatment of RA

Drug	Mechanism of Action	Route of administration	Usual dose	Common adverse reactions
Methotrexate	Inhibition of folate metabolism	Oral, intramuscular, and intravenous	7.5–20 mg/ week	Gastrointestinal discomfort, liver function damage (elevated liver enzymes), stomatitis, alopecia, skin rash, bone marrow suppression can happen occasionally, drug-induced pneumonitis was reported but very rare
Sulfasalazine	The metabolite 5-aminosalicylic acid inhibits prostaglandin, leukotriene synthesis and neutrophil function	Oral	2 ~ 6 g/day, divided into 2 ~ 4 times	Allergic reactions (should not be used in patients allergic to sulfonamides), and gastrointestinal reactions happen occssionally; bone marrow suppression was reported
Leflunomide	Inhibition of pyrimidine synthesis	Oral	10–20 mg/ day as a single dose	Liver function abnormality as elevated liver enzymes, gastrointestinal discomfort, alopecia, skin rash can happen occasionally, drug-induced pneumonitis are rare
Hydroxychloroquine	Stabilizes lysosomal membranes, inhibits multiple enzyme activities, inhibits prostaglandin and interleukin 1 synthesis, and inhibits neutrophils function	Oral	0.2–0.4 g/ day 1–3 times	Allergic reaction, dye fundus lesion
Tripterygium wilfordii Hook II	Not completely eludicated. But may be related with multiple immunosuppressive and antiinflammatory effects	Oral	30–60 mg/ day in 2–3 divided doses	Gonadotoxicity, abnormal liver function tests, gastrointestinal discomfort, skin rash, bone marrow suppression occur occasionally
Iguratimod	Inhibits nuclear factor kappa B (NF-κB) activity, inhibits immunoglobulin synthesis and cyclooxygenase-2 (COX-2)	Oral	50 mg/day in 2 divided doses	Abnormal liver function tests, gastrointestinal discomfort, skin rash happen occasionally

RA, rheumatoid arthritis; NF-κB, nuclear factor kappa B.

There is currently insufficient evidence to support the use of bD-MARDs or tsDMARDs as the first-line therapy. According to existing evidence, the combination of bDMARDs/tsDMARDs are mostly prescribed to patients with poor response or intolerance to csDMARDs. Studies have shown that the combination of MTX with biologics is more effective than MTX alone in MTX - naive RA patients. However, it is lack of evidence that biologic monotherapy is superior to MTX monotherapy.^[85,86]^ We still recommend MTX as the preferred csDMARD for the first-line therapy of treatment-naive RA patients in China taking efficacy, adverse reactions, economy, and accessibility of the medications as well as physicians’ practice experience into consideration.

***Recommendation 7: Short-term glucocorticoid (GCs) should be considered based on disease activity when initiating or changing csDMARDs (2B). Adverse reactions should be closely monitored during GCs treatment. Monotherapy or long-term, high-dose glucocorticoid use is strongly not recommended (1A)***

GCs has strong anti-inflammatory effects and can be used to inhibit acute inflammation in RA. Numerous studies have suggested that the combination of a csDMARD with short-term, low-dose GCs can relieve pain, shorten the duration of morning stiffness, reduce SJC and TJC and improve physical function, quality of life, patient global assessment (PGA) and PtGA in patients with active RA.^[87,88,89]^ GCs could not prevent or delay joint erosion in RA and thus, should not be used alone. Moreover, long-term or high-dose GC therapy is not recommended due to increased risks of various complications such as infections, cardiovascular/cerebrovascular disorders and osteoporosis.^[90,91,92]^ The dose of GCs should not be higher than prednisone 10 mg/day or equivalent. GCs should be tapered and discontinued as early as possible, usually within 6 months. There is no need to continue GCs use in patients on bMDARDs/tsDMARDs. EULAR recommends that GCs should be discontinued as quickly as possible after initiation of bDMARDs/tsDMARDs.^[14]^ Non-steroidal anti-inflammatory drugs (NSAIDs) can be used to relieve pain in RA patients but NSAIDs should be used with caution due to the risk of cardiovascular and gastrointestinal adverse reactions,^[90,93]^ especially in elderly patients and patients with related underlying diseases.

***Recommendation 8: When a single conventional synthetic DMARD (csDMARDs) treatment can’t reach clinical improvement within 3 months or can’t achieve treatment target within 6 months, we recommend to switch to another csDAMRD or combine with another DMARDs (2B); or combine one DMARD with one of the biological/ tsDMARDs (2B);***

After being treated with methotrexate, or leflunomide or sulfasalazine monotherapy, the patients can’*t* reach the treatment target, the current therapy should be adjusted timely. Inadequate response is generally defined as failure to achieve remission or low disease activity within 3 months with less than 50% improvement in composite disease activity criteria, or failure to achieve remission or low disease activity after 6-month treatment. For second-line therapy, there is very limited evidence to clarify the advantages and disadvantages between switching to another csDMARD, combination of csDMARDs and combining one csDMARD with one of the bDMARDs or one of the small molecule tsDMARDs. Limited RCT evidence showed non-significant difference between these treatment regimens.^[94]^ Although EULAR and ACR guidelines conditionally recommend the combination a bDMARD or tsDMARD prior to csDMARD combination, this recommendation is made mainly based on considerations of quicker efficacy onset and drug retention rate, however, the evidence level is low.^[13,14]^ We do not differentiate the priority of these two regimens due to the low evidence level and consideration of China’s national economic conditions and comorbidities, such as viral hepatitis and tuberculosis infection. In addition, this guideline does not specially distinguish the treatment regimens according to the presence of poor prognostic factors or not, though the factors should be considered by clinicians when they formulating treatment regimens. However, the existing evidence suggests no precedence over each other for adjusting csD-MARDs or combining bDMARDs/tsDMARDs in second-line treatment selection of RA based on the poor prognostic factors only.^[13,95]^

If csDMARDs combination therapy is adopted, either two of MTX, sulfasalazine, and leflunomide can be combined; however, if MTX is used in combination with leflunomide, attention should be paid to liver function impairment^[96,97]^ and hematological adverse reactions.^[98]^ Hydroxychloroquine is commonly used in combination regimens due to lower structural efficacy but can also be used alone in patients with low disease activity at the early stage of the disease.^[13,14,99]^ In addition, hydroxychloroquine can improve patients’ glucose and fat metabolism, making itself suitable for patients with concomitant cardiovascular diseases.^[100]^

The efficacy of the herbal medicine Tripterygium wilfordii Hook II in the treatment of RA has been recognized. Studies have shown that the efficacy of Tripterygium wilfordii Hook II monotherapy is not inferior to MTX monotherapy in the treatment of RA, and Tripterygium wilfordii Hook II in combination with MTX or tumor necrosis factor α (TNFα) inhibitor also has good efficacy and safety.^[101,102]^ Tripterygium wilfordii Hook II can be used as csDMARD in addition to MTX, sulfasalazine, and leflunomide, but they are contraindicated in patients who are preparing for pregnancy, in pregnancy, in lactation, or with fertility requirements because of their genital toxicity. Although a few reports have shown better efficacy of total glucosides of paeony in combination with csDMARD,^[103,104]^ its efficacy in RA treatment needs further investigation.

Iguratimod, an anti-rheumatic drug developed in China with characteristics of csDMARDs, has been used in the treatment of RA in China and some Asian countries. There is evidence that iguratimod plus MTX is superior to MTX alone and has a good safety profile,^[105,106,107]^ so it can be used as a second-line therapy for RA.

Tumor necrosis factor-alpha (TNF-a) inhibitors are the most widely used biological DMARDs in treating RA with abundant evidence. Several TNFα inhibitors have been launched in China including the monoclonal antibody drugs adalimumab, infliximab, golimumab and certolizumab, as well as the receptor fusion protein drug etanercept, all of which have been demonstrated to be efficacious and safe in the treatment of RA with sufficient evidence.^[108,109,110,111,112,113,114,115,116,117,118,119]^ We recommend combining TNFα inhibitors with csDMARD.^[120,121,122,123]^ For patients receiving treatment with TNFα inhibitors, special attention should be paid to the risk of hepatitis virus and Mycobacterium tuberculosis infections or reactivation of pre-existing infections. Patients should be screened prior to the treatment with TNFα inhibitors and be monitored regularly during treatment.^[124,125,126]^ Pre-treatment screening should include hepatitis B virus (HBV) and hepatitis C virus (HCV) serology tests (including HBV antigen and anti-HBV antibodies, anti-HCV antibody, and viral load test should be considered if necessary); Purified protein derivative (PPD) skin test and/or interferon (IFN)-γ release assay (T-SPOT. TB or QuantiFERON-TB GOLD) should be selected according to local accessibility, chest radiograph (X-ray or CT should be chosen according to local medical technological availability and patient’s conditions).^[126]^ For patients with hepatitis virus infection and latent mycobacterium tuberculosis infection, prophylaxis treatment should be administered. The prophylaxis treatment should follow the recommendations from infectious disease experts and be adjusted based on the patient’s situation.^[15,126]^

Evidence have proven the efficacy and safety of tocilizumab, a recombinant humanized IgG1 monoclonal antibody that targeted interleukin-6 (IL-6) receptors, in the treatment of RA. Recent research have shown that tocilizumab monotherapy can also achieve good clinical efficacy.^[127,128,129,130,131]^ For RA patients who are intolerant to conventional synthetic DMARDs, tocilizumab monotherapy should be considered.

Abatacept is a T-cell co-stimulatory inhibitor that inhibits T cell activation by specifically interfering with CD28 interaction with CD80/86.^[132]^ Many evidence have supported the efficacy and safety of abatacept in treating RA^[133,134,135]^ and it can be used as one of the bDMARD medications.

JAK inhibitors are a class of tsDMARDs targeted at the JAK-STAT signaling pathway. At present, JAK inhibitors approved in China include tofacitinib, baricitinib, and upadacitinib. Current research evidence have shown that JAK inhibitors have good efficacy and safety in RA,^[136,137,138,139,140,141,142,143,144]^ but it should be noted that such drugs may increase the risk of major cardiovascular events (MACEs), malignancies and venous thrombosis.^[145,146,147,148]^ The following risk factors for MACE and malignancies must be taken into consideration when prescribing a JAK-inhibitor: age over 65 years, history of current or past smoking, other cardiovascular risk factors (such as diabetes, obesity, hypertension), other risk factors for malignancy (current or previous history of malignancy), risk factors for thromboembolic events (history of myocardial infarction or heart failure, cancer, inherited coagulation disorders or a history of thrombosis, as well as patients taking combined female hormonal contraceptives or hormone replacement therapy, undergoing major surgery or immobile).^[14,149]^ These risk factors should be adequately assessed prior to treatment with JAK inhibitors and be monitored regularly.

Rituximab, an anti-CD20 monoclonal antibody, has been shown to be effective in the treatment of RA.^[150,151,152]^ It may serve as an alternative for patients with RA who show inadequate response or intolerance to biologics and JAK inhibitors.

Based on the existing evidence, no precedence over each other for TNF-a inhibitors, tocilizumab and tofacitinib in the treatment of RA.^[153]^ If a patient failed bDMARD or tsDMARD, treatment with another bDMARD or tsDMARD should be considered. Switching to another TNFα inhibitor, tocilizumab, rituximab, or JAK inhibitor have been proved to be effective after failure of one of the TNFα inhibitors.^[154,155,156,157,158]^ However, the efficacy of switching to another JAK inhibitor after failure of one JAK inhibitor is uncertain. For patients who failed tocilizumab or one JAK inhibitor may be treated with an agent with different mode of action. bDMARDs and tsDMARDs are associated with increased risk of infections compared with csDMARDs.^[159,160,161]^ Therefore, for all patients receiving bD-MARDs or tsDMARDs, special attention should be paid not only to the aforementioned adverse events related to TNFα inhibitors and JAK inhibitors, but also to the risk of various infections, especially respiratory tract infections (including influenza virus, *Streptococcus pneumoniae*, *etc*.) and herpes zoster. Vaccination should be considered for patients without contraindications.^[126,162,163]^

Biosimilars have the same mechanism as original biologics with lower cost, so with increased accessibility to biologics for patients.^[164]^ A variety of biosimilars have got approved in China. According to the results of a meta-analysis including 27 RCTs, the efficacy and safety of the approved biosimilars are not significantly different from those of the originals in the treatment of RA.^[165]^ The efficacy and safety of biosimilars have been endorsed by 2021 American College of Rheumatology guideline for the Treatment of RA^[13]^ and EULAR recommendations for the management of RA with synthetic and biological DMARDs: 2022 update.^[14]^

Intra-articular injection of GCs or etanercept can improve symptoms in patients with single affected joints,^[166]^ however, overuse of this procedure should be avoided. Intra-articular injection should be applied with caution to the risk of secondary infections related to joint cavity puncture. Studies have demonstrated that technetium(99Tc) methylenediphosphonate (99Tc-MDP) may be beneficial in the treatment of RA,^[167,168]^ but further research is needed.

Most RA patients can reach disease remission or low disease activity through standard care. However, some patients still have active disease even after being treated with standard treatment. These patients are defined as “refractory or difficult-to-treat (D2T) RA”, accounting for 5%-20% of total RA patients.^[169,170]^ The following three criteria were agreed by all Task Force members of EULAR as mandatory elements of the definition of difficult-to-treat RA: (1) Treatment according to European League Against Rheumatism (EULAR) recommendation and failure of ≥2 biological disease-modifying antirheumatic drugs (DMARDs) /targeted synthetic DMARDs (with different mechanisms of action) after failing conventional synthetic DMARD therapy (unless contraindicated); (2) presence of at least one of the following: a, at least moderate disease activity (*e.g*., DAS28-ESR > 3.2 or CADI > 10); b, Signs (including acute phase reactants and imaging) and/or symptoms suggestive of active disease (joint related or other); c, inability to taper GCs treatment ( < 7.5 mg/day prednisone or equivalent); d, rapid radiographic progression (with or without signs of active disease); e, Well-controlled disease according to above standards, but still having RA symptoms that are causing a reduction in quality of life; and (3) the management of signs and/or symptoms is perceived as problematic by the rheumatologist and/or the patient.^[169,171]^ The factors contributing to D2T include drug ineffectiveness or presence of factors for adverse drug reactions (*e.g*., smoking, obesity, patient’s genetic and immunologic background), as well as comorbidities and other factors affecting the clinical outcome (*e.g*., interstitial pneumonia and fibromyalgia).^[172]^ Multivariate analysis identified high RF levels, DAS28-ESR, and coexisting pulmonary disease as predictive risk factors of D2T RA.^[170]^ For such patients, the factors contributing to D2T should be fully evaluated, and individualized treatment regimens should be formulated.^[173,174]^

***Recommendation 9: Tapering of DMARDs (bDMARDs/ tsDMARDs or csDMARDs) could be considered if the patient reached disease remission for at least 6 months. Close monitoring of disease activity during tapering is warranted given the potential for disease flare. (2C). For patients treated with DMARDs combination therapy, if the patient is in sustained remission after tapering of one DMARD, then this drug could be discontinuation (2C)***

Based on the current evidence, dose reduction of DMARDs can be considered for RA patients in sustained remission, but this is only considered as an option rather than a recommendation, and patients with dose reduction should be closely monitored.^[13,14,175,176]^ Currently the specific duration of “sustained remission” is still inconclusive, and a systemic review shows that a 6-month remission period may be appropriate.^[175]^ The 2021 ACR guidelines also recommend that tapering of DMARDs should only be started if a patient is in persistent stringent remission for at least 6 months to ensure stable disease control.^[13]^ For patients receiving csDMARD + b/tsDMARD combination therapy, whether csDMARDs or b/ tsDMARDs should be tapered first is still under debate.^[177,178,179]^ Considering that discontinuation of all DMARDs is associated with moderate to high risk of RA flare and potentially irreversible damages in most patients, we recommend that patients maintain at least 1 DMARD rather than discontinue all DMARDs.^[180,181]^ It is still controversial whether DMARDs can be tapered in RA patients who achieve low disease activity without remission.

***Recommendation 10: RA patients should be educated on the nature, course, treatment, and self-management of the disease and psychological support should also be provided (1A). Patients with RA should be advised to lifestyle modification, including smoking cessation, weight control, healthy diet and exercise (1A)***

Patient education is essential for disease management, because it helps to improve the effectiveness of RA treatment. Clinicians should help patients to fully understand the nature and prognosis of RA, to help them to set up the confidence to receive standard treatment, and to remind them to be monitored and followed-up regularly and facilitate patients to take appropriate self-management measures.^[182,183,184,185]^ Health education can also provide guidance on smoking cessation, weight control, healthy diet, and appropriate exercise, helping patients improve their lifestyle. Compared with the general population, RA patients have increased incidences of anxiety and depression, and RA patients with anxiety and/ or depression tend to have worse clinical outcomes.^[186,187,188]^ Studies have shown that positive, effective cognitive intervention and psychological support are significantly helpful for relieving pain and improving physical function, mental health, and disease activity in RA patients.^[189,190]^ Smoking is closely related to the onset and progression of RA, the efficacy of drug therapy, and risk of interstitial lung disease, cardiovascular diseases, osteoporosis, and tumors.^[191,192,193]^ Thus all RA patients should quit smoking. Obese people have an increased risk of RA ^[194,195]^ and obesity have an adverse impact on the disease activity and adverse drug reactions of RA.^[196,197]^ Weight control can help RA patients improve disease activity and prognosis.^[198,199,200]^ A healthy diet helps reduce inflammation and alleviate symptoms in RA patients.^[201,202,203,204,205]^ Appropriate exercise and physical therapy (*e.g*., aerobic exercise, resistance exercise, and functional exercise) can enhance the flexibility and stability of the joint and improve the patients’ symptoms, physical function, and quality of life.^[206,207,208,209,210,211,212]^

This guideline provides recommendations for important clinical issues on the diagnosis, evaluation, treatment, and follow-up of RA based on the existing evidence published domestic and abroad, as well as the disease characteristics and medical conditions of RA and the practical experience of rheumatologists in China. Rheumatologists and physicians in other medical specialty engaged in the diagnosis and treatment of RA should carry out standardized diagnosis and treatment referring to this guideline, so as to ensure the quality of treatment, improve the diagnosis and treatment of RA in China, and improve the prognosis of patients. However, in view of the individual differences in RA, it is necessary to fully consider patients’ specific situation and develop individualized diagnosis and treatment regimens through doctor-patient joint decision-making in clinical practice. In addition, the existing evidence is insufficient to provide clear answers to some important clinical issues in the diagnosis and treatment of RA, such as how to identify patients with poor response to csDMARDs, how to early identify D2T patients and provide effective treatment accordingly. Further studies are needed to further improve the treatment effect and prognosis of RA.

**Figure 1:**
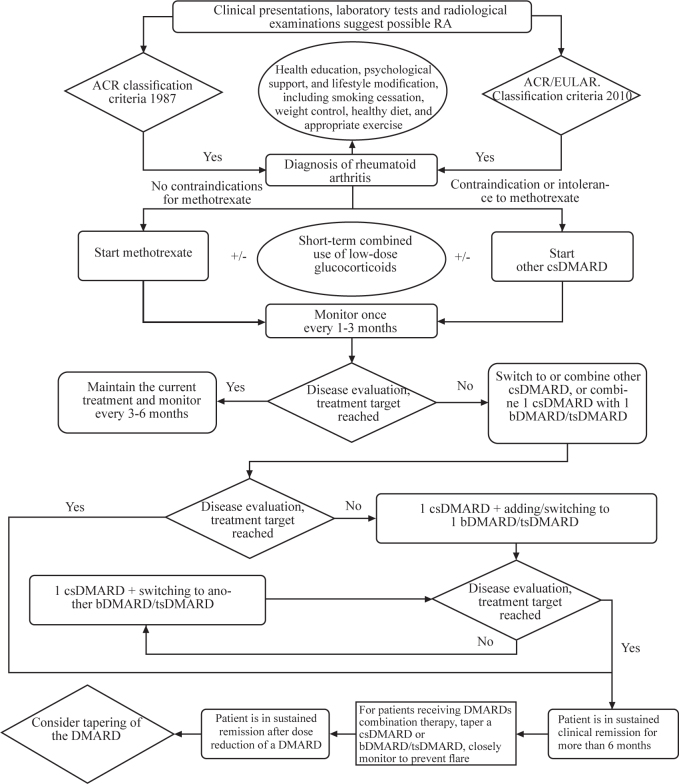
Recommended RA diagnosis and treatment process.

## Methodology Panel

Yaolong Chen (Evidence-Based Social Science Research Centre, School of Public Health, Lanzhou University, Lanzhou University GRADE Center).

## Drafting Group

Nan Jiang (Department of Rheumatology and Clinical Immunology, Peking Union Medical College Hospital, Peking Union Medical College); Dagula (Department of Rheumatology, The Affiliated Hospital of Inner Mongolia Medical University); Shuang Liu (Department of Rheumatology and Immunology, First Affiliated Hospital of Kunming Medical University); Yaqian Liu (Department of Rheumatology and Immunology, the First Affiliated Hospital of Anhui Medical University); Jianda Ma (Department of Rheumatology and Immunology, Sun Yat-Sen Memorial Hospital, Sun Yat-Sen University); Xiaofei Peng (Department of Rheumatology and Immunology.

The Second Xiangya Hospital of Central South University); Sun Yiduo (Department of Rheumatology and Immunology, The First Affiliated Hospital, Zhejiang University School of Medicine); Suli Wang (Department of Rheumatology and Immunology, Renji Hospital Affiliated to Shanghai Jiao Tong University School of Medicine).

## Evidence Grading Panel

Xufei Luo (Evidence-Based Social Science Research Centre, School of Public Health, Lanzhou University, Lanzhou University GRADE Center); Ye Wang (Evidence-Based Social Science Research Centre, School of Public Health, Lanzhou University, Lanzhou University GRADE Center); Haodong Li (Evidence-Based Social Science Research Centre, School of Public Health, Lanzhou University, Lanzhou University GRADE Center); Renfeng Su (Evidence-Based Social Science Research Centre, School of Public Health, Lanzhou University, Lanzhou University GRADE Center).

## Expert Panel

Guoqiang Chen (Department of Rheumatology and Immunology, The First People’s Hospital of Foshan); Yaolong Chen (Evidence-Based Social Science Research Centre, School of Public Health, Lanzhou University, Lanzhou University GRADE Center) ; Chen Zhen (Department of Rheumatology and Immunology, The Second Affiliated Hospital of Fujian Medical University); Lie Dai (Department of Rheumatology, Sun Yat-Sen Memorial Hospital, Sun Yat-Sen University);; Feng Ding (Department of Rheumatology, Qilu Hospital of Shandong University); Xinwang Duan (Department of Rheumatology and Immunology, The Second Affiliated Hospital of Nanchang University); Yongfei Fang (Rheumatology and Immunology Department of Traditional Chinese Medicine, The Southwest Hospital of Army Medical University); Jieruo Gu (Department of Rheumatology and Immunology, the Third Affiliated Hospital, Sun Yat-Sen University); Dongyi He (Department of Rheumatology, Shanghai Guanghua Hospital of Integrated Traditional Chinese and Western Medicine); Lan He (Department of Rheumatology and Immunology, the First Affiliated Hospital of Xi’an Jiaotong University); Cibo Huang (Department of Rheumatology, South China Hospital, Health Science Center); Wenhui Huang (Department of Rheumatology and Immunology, Second Affiliated Hospital, Guangzhou Medical University); Lindi Jiang (Department of Rheumatology, Zhongshan Hospital, Fudan University); Zhenyu Jiang (Department of Rheumatology and Immunology, The First Hospital of Jilin University);); Caifeng Li (Department of Rheumatology, National Center for Children’s Health, Beijing Children’s Hospital, Capital Medical University); Fen Li (Department of Rheumatology and Immunology, The Second Xiangya Hospital of Central South University); Hongbin Li (Department of Rheumatology, The Affiliated Hospital of Inner Mongolia Medical University); Mengtao Li (Department of Rheumatology and Clinical Immunology, Peking Union Medical College Hospital, Chinese Academy of Medical Sciences & Peking Union Medical College); Xiao Li (Department of Rheumatology, The Second Hospital of Shanxi Medical University); Xiaomei Li (Department of Rheumatology, The First Affiliated Hospital of University of Science and Technology of China); Xiaoxia Li (Department of Rheumatology and Allergy, Xuanwu Hospital, Capital Medical University); He Lin (Department of Rheumatology and Immunology, Fujian Provincial Hospital); Jin Lin (Department of Rheumatology, The First Affiliated Hospital, Zhejiang University School of Medicine); Dongzhou Liu (Department of Rheumatology and Immunology, Shenzhen People’s Hospital); Shengyun Liu (Department of Rheumatology and Immunology, The First Affiliated Hospital of Zhengzhou University); Yi Liu (Department of Rheumatology and Immunology, West China Hospital, Sichuan University); Hui Luo (Department of Rheumatology and Immunology, West China Hospital, Sichuan University); Liangjing Lu (Department of Rheumatology and Immunology, Renji Hospital Affiliated to Shanghai Jiao Tong University School of Medicine); Li Ma (Department of Rheumatology, China Japan Friendship Hospital);; Yifang Mei (Department of Rheumatology and Immunology, Shenzhen Third People’s Hospital); Haili Shen (Department of Rheumatology, Lanzhou University Second Hospital); Zongwen Shuai (Department of Rheumatology and Immunology, The First Affiliated Hospital of Anhui Medical University); Hui Song (Department of Rheumatology, Jishuitan Hospital, Beijing); Lingyun Sun (Department of Rheumatology, Nanjing Drum Tower Hospital of Nanjing University Medical School); Yin Su (Department of Rheumatology, Peking University People’s Hospital); Xinping Tian (Department of Rheumatology and Immunology, Peking Union Medical College Hospital); Caihong Wang (Department of Rheumatology and Immunology, The Second Hospital of Shanxi Medical University); Guochun Wang (Department of Rheumatology, China Japan Friendship Hospital); Jibo Wang (Department of Rheumatology & Clinical Immunology, Affiliated Hospital of Qingdao University);; Qian Wang (Department of Rheumatology and Immunology, Peking Union Medical College Hospital, Chinese Academy of Medical Sciences); Yongfu Wang (Department of Rheumatology, the First Affiliated Hospital of Baotou Medical College);; Youlian Wang (Department of Rheumatology and Immunology, Jiangxi Provincial People’s Hospital); Wei Wei (Department of Rheumatology and Immunology, Tianjin Medical University General Hospital); Huaxiang Wu (Department of Rheumatology and Immunology, the Second Affiliated Hospitalvof Zhejiang University, School of Medicine); Lijun Wu (Department of Rheumatology and Immunology, People’s Hospital of Xinjiang Uygur Autonomous Region);; Zhenbiao Wu (Department of Rheumatology and Immunology, Tangdu Hospital, the Second Affiliated Hospital of Air Force Medical University); Huji Xu (Department of Rheumatology, the Second Military Medical University Changzheng Hospital); Jian Xu (Department of Rheumatology and Immunology, First Affiliated Hospital of Kunming Medical University); Chengde Yang (Department of Rheumatology and Immunology, Ruijin Hospital, Shanghai Jiao Tong University School of Medicine); Min yang (Department of Rheumatology and Immunology, Nanfang Hospital, Southern Medical University); Pingting Yang (Department of Rheumatology and Immunology, The First Affiliated Hospital of China Medical University); Xiaofeng Zeng (Department of Rheumatology and Clinical Immunology, Peking Union Medical College Hospital, Chinese Academy of Medical Sciences & Peking Union Medical College); Feng Zhan (Department of Rheumatology and Immunology, Hainan Provincial People’s Hospital); Fengxiao Zhang (Department of Rheumatology and Immunology, Hebei General Hospital); Miaojia Zhang (Department of Rheumatology, The First Affiliated Hospital of Nanjing Medical University); Wen Zhang (Department of Rheumatology and Immunology, Peking Union Medical College Hospital, Chinese Academy of Medical Sciences); Xiao Zhang (Department of Rheumatology and Immunology, Guangdong Provincial People’s Hospital); Xuewu Zhang (Department of Rheumatology and Immunology, Peking University People’s Hospital); Zhiyi Zhang (Department of Rheumatology and Immunology, The First Affiliated Hospital of Harbin Medical University); Zhuoli Zhang (Department of Rheumatology, the First Hospital of Peking University); Dongbao Zhao (Department of Rheumatology and Immunology, Changhai Hospital Affiliated to Naval Medical University); Yan Zhao (Department of Rheumatology and Clinical Immunology, Peking Union Medical College Hospital, Chinese Academy of Medical Sciences & Peking Union Medical College); Yi Zheng (Department of Rheumatology and Immunology, Beijing Chaoyang Hospital Affiliated to Capital Medical University); Zhaohui Zheng (Department of Rheumatology and Immunology, First Affiliated Hospital of Air Force Medical University); Jing Zhu (Department of Rheumatology and Immunology, Sichuan Provincial People’s Hospital); Hejian Zou (Department of Rheumatology and Immunology, Huashan Hospital Affiliated to Fudan University).
